# Manhattan++: displaying genome-wide association summary statistics with multiple annotation layers

**DOI:** 10.1186/s12859-019-3201-y

**Published:** 2019-11-27

**Authors:** Christopher Grace, Martin Farrall, Hugh Watkins, Anuj Goel

**Affiliations:** 1 Division of Cardiovascular Medicine, Radcliffe Department of Medicine, John Radcliffe Hospital, University of Oxford, Oxford, OX3 9DU UK; 2 Wellcome Centre for Human Genetics, University of Oxford, Oxford, OX3 7BN UK

**Keywords:** Manhattan plot, GWAS, Meta-analysis, R, Software, CRAN

## Abstract

**Background:**

Over the last 10 years, there have been over 3300 genome-wide association studies (GWAS). Almost every GWAS study provides a Manhattan plot either as a main figure or in the supplement. Several software packages can generate a Manhattan plot, but they are all limited in the extent to which they can annotate gene-names, allele frequencies, and variants having high impact on gene function or provide any other added information or flexibility. Furthermore, in a conventional Manhattan plot, there is no way of distinguishing a locus identified due to a single variant with very significant *p*-value from a locus with multiple variants which appear to be in a haplotype block having very similar *p*-values.

**Results:**

Here we present a software tool written in R, which generates a transposed Manhattan plot along with additional features like variant consequence and minor allele frequency to annotate the plot and addresses these limitations. The software also gives flexibility on how and where the user wants to display the annotations. The software can be downloaded from CRAN repository and also from the GitHub project page.

**Conclusions:**

We present a major step up to the existing conventional Manhattan plot generation tools. We hope this form of display along with the added annotations will bring more insight to the reader from this new Manhattan++ plot.

## Background

A Manhattan plot, which plots the association statistical significance as –log10(*p*-value) in the y-axis against chromosomes in the x-axis, is a good way of displaying millions of genetic variants in one figure. One can easily spot regions of the genome that cross a particular significance threshold. Furthermore, it makes it easy to identify regions that can be taken forward for replication. Several software packages (QQMAN [1], GWAMA [2], IGV [3], https://genome.sph.umich.edu/wiki/Code_Sample:_Generating_Manhattan_Plots_in_R, SNPEVG [4]) come bundled with a plotting feature or a small R script which can generate a Manhattan plot. These scripts generate the plot but because of the lack of any further information in the plot (annotating the plot with gene names, identifying how significant are low frequency variants and high impact consequence variants in the GWAS), the Manhattan plot is losing its importance in more recent GWAS publications. However, with availability of large cohorts (eg. UK Biobank) and power to detect more loci crossing genome wide significant threshold (over 500 in the recent Blood Pressure GWAS [5]), it is a tedious, time-consuming process to annotate gene names manually on a Manhattan plot. Another drawback with the conventional plot is the inability to identify the number of variants hiding behind “a” visible dot. In order to overcome the limitation to annotate ever-increasing loci discovered, researchers have started transposing [6–11] the Manhattan plot to give more room to display the gene names on the plot. Manhattan++ software tool reads the genome-wide summary statistic on millions of variants and generates the transposed Manhattan++ plot with user defined annotations like gene-names, allele frequencies, variant consequence and summary statistics of loci of interest.

## Implementation

**Fig. 1:**
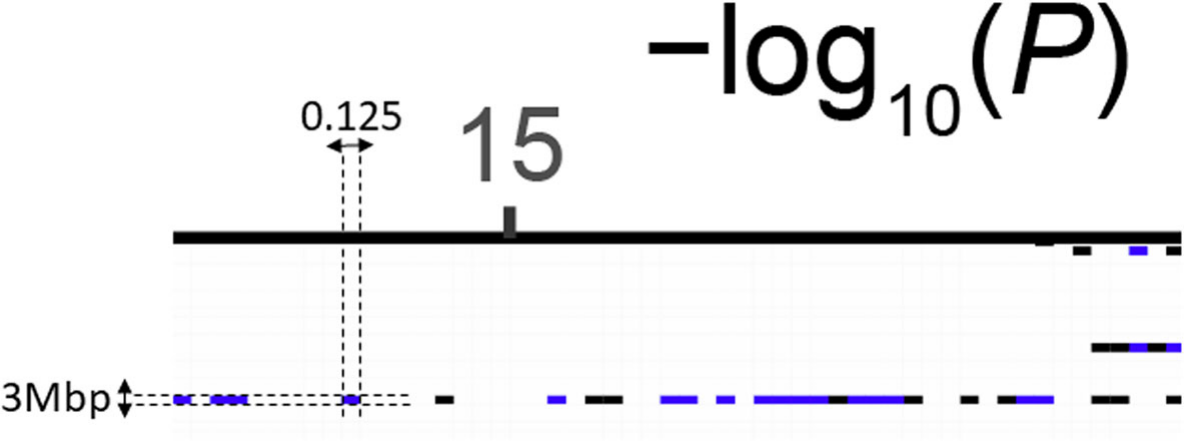
Displaying the dimensions of the matrix. The genome is split into chunks of 3 Mbp and the association statistics in chunks of 0.125 –log10(p). The number of variants in each cell of the matrix is counted and stored in memory. The colour of the cell is assigned according to the config file settings

The software is written in R (version > = 3.4.0) and requires ggplot2, ggrepel, reshape2 and gridExtra packages (along with their dependencies). The software needs three files as input. The first file contains the genome-wide summary statistics. This file should contain the variant name, chromosome, position, *p*-values, minor allele frequency (MAF) and consequence. The code can cope with different column header names and accept compressed summary statistics file. The second file contains the information on the loci of interest that are to be annotated on the plot. This file contains lead sentinel variant name, chromosome, position, effect allele frequency, odds ratio, *p*-value, novel/known and gene-name for the loci of interest. The third file is a configuration file that instructs the software on the colours, bin sizes and annotation features required for display (Table 1). The display consists of two panels where the left panel is used for transposed Manhattan plot and the right panel to display information on the loci of interest. The script splits the genome (Fig. 1) into user-defined chunks (default 3 million base pairs (Mbp)) and association *p*-values chunks (default –log10 *p*-value of 0.125) and creates an empty matrix. The script reads the summary statistics and increments the counter for the respective bin where the variant lies in the matrix. Variants which have a *p*-value<1e-20 (default) are assigned *p*-value = 1e-20 and the bin count is incremented accordingly and limited information is lost. The bin count matrix is then used by ggplot2 to display the heatmap using the colours as defined in the configuration file. All the parameters can be edited in the configuration file and when calling the function in R according to user preference.

**Table 1:** Relevant columns in the configuration file for the software

idx	Min count	maf	conseq	col	report	Description
1	1	FALSE	FALSE	black	FALSE	Cells with one variant are black.
2	1	FALSE	TRUE	light pink	TRUE	Cells with one variant with high conseq are light pink.
3	1	TRUE	FALSE	green	FALSE	Cells with one variant with MAF less than threshold are green.
4	1	TRUE	TRUE	dark magenta	TRUE	Cells with one variant with MAF less than threshold and high conseq are dark magenta.
5	2	FALSE	FALSE	blue	FALSE	Cells with 2 or more variants are blue.
6	2	FALSE	TRUE	pink	TRUE	Cells with 2 or more variants with high conseq in at least one are pink.
7	2	TRUE	FALSE	red	FALSE	Cells with 2 or more variants with a MAF less than threshold in at least one are red.
8	2	TRUE	TRUE	cyan	TRUE	Cells with 2 or more variants with at least one variant with MAF less than threshold and at least one variant with the conseq flag are cyan.

**Fig. 2:**
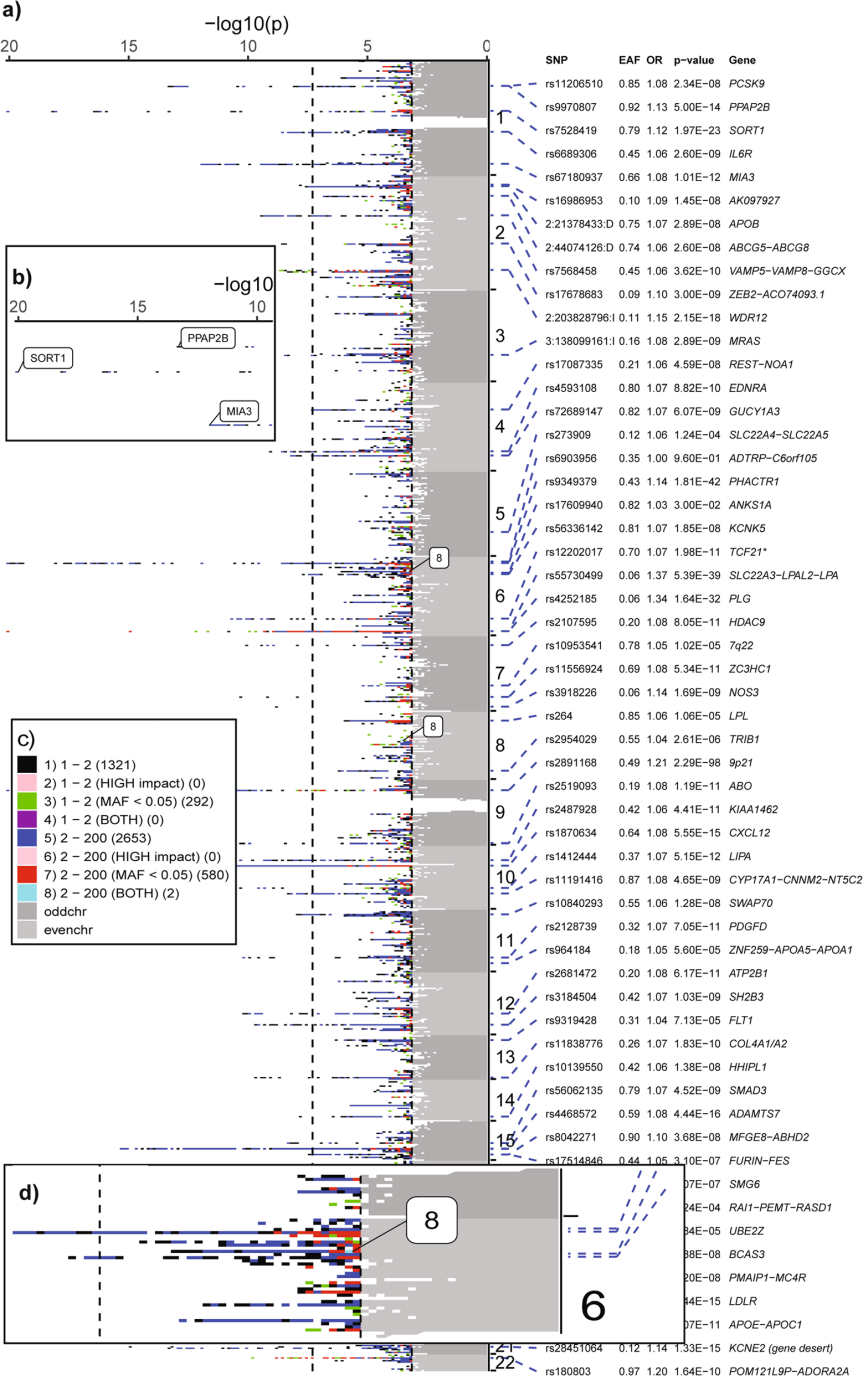
Screenshots of the output from Manhattan++ software. **a**) Transposed Manhattan plot on the left and lead variant annotation on the right. Alternating dark and light grey blocks represent the odd and even chromosomes respectively along the y-axis for those variants have association *p*-value greater than user-defined significance (5% False Discovery Rate). Blocks that contain variants with high impact and/or low MAF are highlighted using a bubble (eg 8). **b**) Zoomed in screenshot showing peak loci names in bubbles. **c**) The key showing the index (1–8), variant count (1, 2–200) in each block, annotation (MAF, impact, both), counter showing number of blocks in the plot for this index (eg. there are 2 blocks having index 8 on chromosomes 6 & 8). **d**) Zoomed in screenshot of a signal where there is a blue block (index 8) that could contain 2–200 variants with at least one variant having low MAF and high impact or two variants, one having low MAF and the other having high impact annotation

Each display cell shows two annotation features (MAF & consequence (conseq)). Report column instructs the code whether a bubble is drawn on the plot (Fig. 2d). These are features of interest like low MAF or high consequence or both. Reporting of bubbles take place on cells which are above the FDR threshold (Fig. 2a). Rest of the cells are alternating dark and light grey blocks represent the odd and even chromosomes respectively. Min.count contains the minimum number of variants in each cell. First 4 rows show configuration for cells with one variant. The next 4 rows are for cells that contain 2 or more variants

## Results

**Fig. 3:**
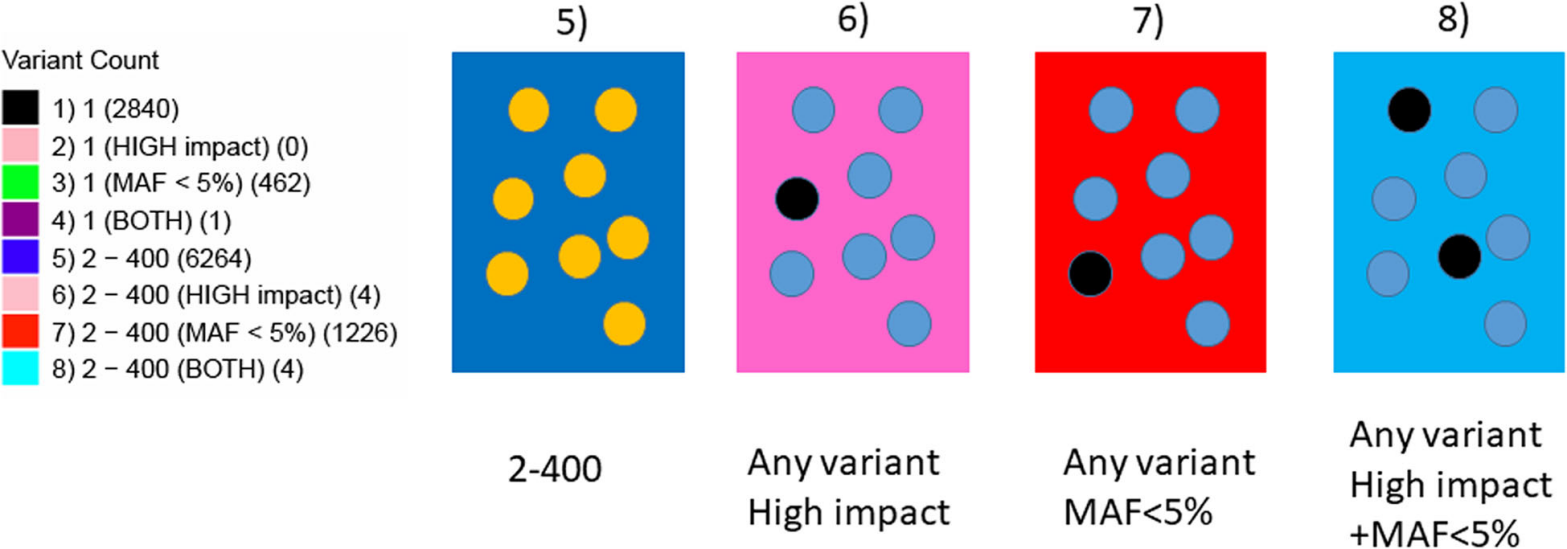
The key. Using the default settings, the Manhattan++ plot generates a key for the plot. For keys #5–8, there are 2–400 variants per cell in the matrix (using different configuration file from Fig. 2). The software calculates the maximum count based on the overall matrix. For key #5, there are 6264 cells in the plot. Key #6 tells us that the cell contains 1 or more high impact variant(s) and there are 4 such cells in the plot. Key #7 gives us information on those cells where there are one or more variant(s) with MAF < 5% and there are 1226 such cells. Key #8 tells us that in 2–400 variants, there is one variant with MAF < 5% and is also high impact. If the cell contains 2 variants, one with MAF < 5% and one that is high impact, then the cell will also get key #8

The software is customizable and can generate a Manhattan of about 20 million variants on a 32 bit desktop personal computer using under 3.4 GB RAM and similar in Linux (Centos 7). The software has been tested to display annotation of 130 loci in a tabular format (Fig. 2a) with odds ratio, effect allele frequency, *p*-value & gene-name. If the number of loci to display goes beyond 130, then we recommend using just the gene (or variant) names and the software will display the names in a force directed manner (Fig. 2b). The colours and the number of variants in each bin are customizable (Table 1, Figs. 2c, 3). This gives the reader an insight into the locus whether it is driven by a single variant (Fig. 2a, *NOS3* locus), variants with low MAF (< 5%) or variants having a “high” impact functional annotation. (Example: Chromosome 6 has a blue block displayed as “8” which denotes that there are multiple (2–200) variants with at least one variant having low MAF and high impact or two variants one having low MAF and the other having high impact annotation as shown in Fig. 2d). The user have the option to save the output as a PDF or a high-resolution TIFF file.

## Conclusions

Here we present the Manhattan++ software which is a major step up from existing tools and addresses the highlighted limitations. Furthermore, the code is customizable and being open source increases the potential for future feature enhancements by the community. We recognize that there are existing scripts that generate a Manhattan plot but none can perform the tasks we have implemented in this software. However, only a handful of them annotate the plot with minimal level of detail (Additional file 1: Supplementary Note, Table S1). Most existing scripts generate a graph in a landscape orientation, which is not enough with ever-increasing number of discovered GWAS loci. A limitation with our method is that it takes one full A4 page of the journal to display but with more researchers reading publications online, this figure is highly web readable and useful for poster presentations. This software adds a lot of information to the existing Manhattan plot and we hope that the readers will be able to derive more information by looking at the Manhattan++ plot.

## Supplementary information

**Additional file 1: **Supplementary Note. **Table S1.**Comparison of the functionalities between existing Manhattan software tools and Manhattan

## Data Availability

Package (manhplot) available via CRAN repository. The software and datasets used during the current study are also available in the GitHub repository, https://github.com/cgrace1978/manhplot.

## Abbreviations

GWAS: Genome wide association study
MAF: Minor allele frequency
Mbp: Million base pairs
