# Supplementary material for: Manhattan++: displaying genome-wide association summary statistics with multiple annotation layers
Source: BMC Bioinformatics. 2019 Nov 27;20:610. doi: 10.1186/s12859-019-3201-y (PMC6882345; doi:10.1186/s12859-019-3201-y)
Supplement: Supplementary file 1 — Additional file 1: Supplementary Note. Table S1.Comparison of the functionalities between existing Manhattan software tools and Manhattan [file 12859_2019_3201_MOESM1_ESM.docx]

**SUPPLEMENTARY NOTE**

1. **Generation of High impact variant consequence**

We used ENSEMBL Variant Effect Predictor (VEP) software (<http://www.ensembl.org/info/docs/tools/vep/index.html> ) to assign every variant a binary flag based on whether it was annotated with one of the “high impact” classifiers as described in VEP documentation (<http://www.ensembl.org/info/genome/variation/prediction/predicted_data.html> ). One could use other variant annotation software packages to generate “high impact” consequence classifiers.

1. **Preparing snpfile**

We have provided a Perl utility script in our GitHub page that can parse the summary statistics file to prepare a loci information (snpfile). The script calls a locus if the p-value is beyond a certain threshold and the variant is certain distance away from the adjacent locus. The gene name assigned to a variant are proximity based. There are 2 gene databases that we have provided in our GitHub page for hg19 & hg38 from PLINK website (https://www.cog-genomics.org/plink/1.9/resources#genelist) but as long as the gene database is formatted accordingly, the script can cope with future builds too assuming the summary statistics also are from that genome build. The analyst still need to add a column describing which locus is known/novel.

1. **Existing software packages and their output**

Manhattan plots generated from existing software packages/script. The data plotted is the same as which was used to plot in Figure 2 (from the CARDIoGRAMplusC4D 2015 Nature Genetics summary statistics). This data can be accessed from the GIT Hub project page.

1. <https://genome.sph.umich.edu/wiki/Code_Sample:_Generating_Manhattan_Plots_in_R>


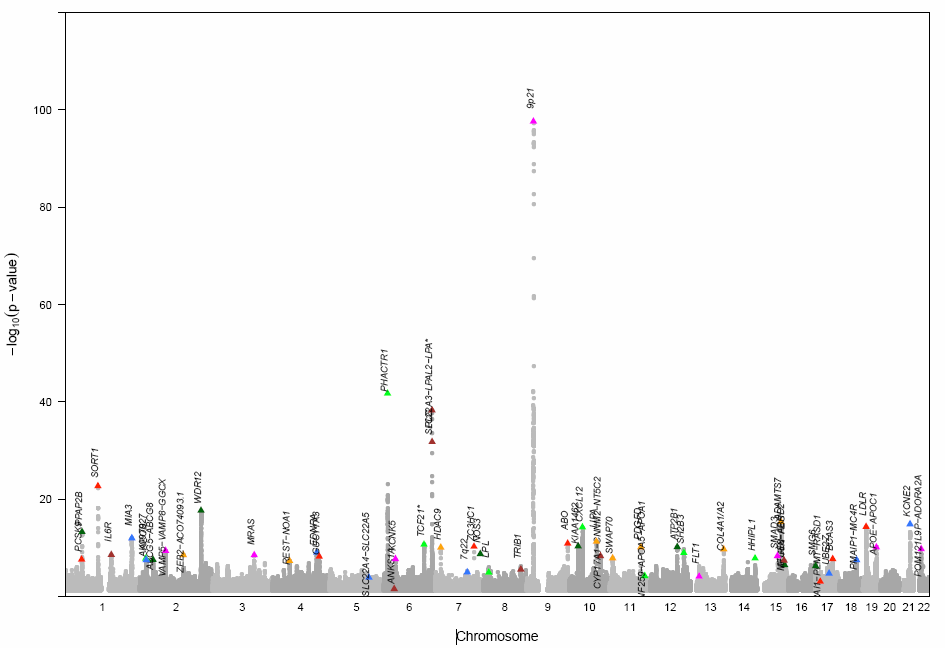


1. QQMAN:


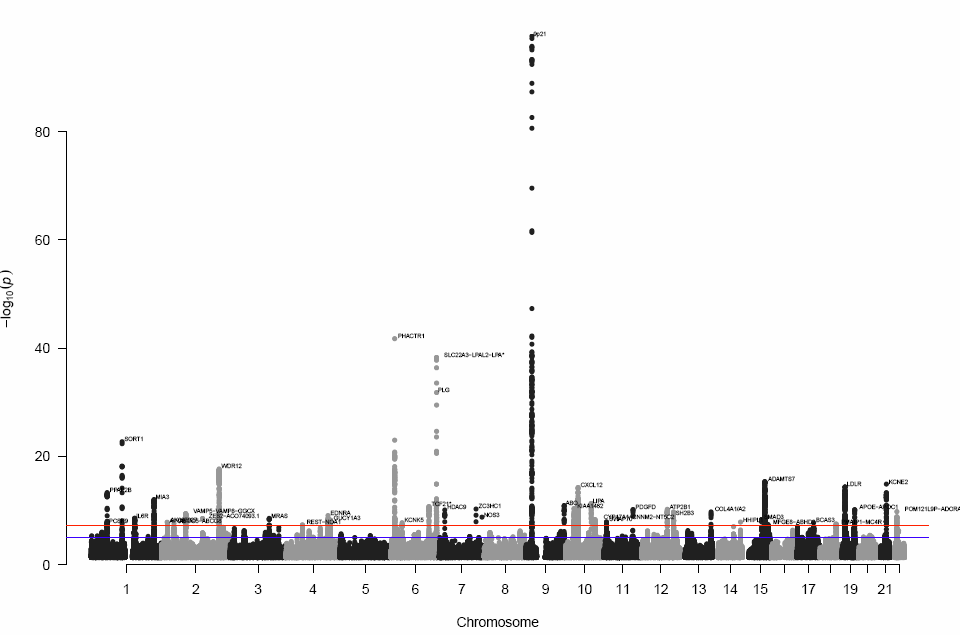


1. **Comparison of existing Manhattan software with Manhattan++**

Supplementary Table 1. Comparison of the functionalities between existing Manhattan software tools and Manhattan++.

|  | UMich | QQMAN | GWAMA | Manhattan++ |
| --- | --- | --- | --- | --- |
| Software language | R | R | R | R |
| Software version number (date where version not available) | Sep-2019 | 0.1.4 | Sep-2019 | 1.0 |
| Gene name | Yes | Yes | No | Yes |
| High resolution vector graphics output (PDF) | Yes | Yes | Yes | Yes |
| Flexibility on positioning of gene name (by known/novel) | No | No | No | Yes |
| Capability to use external variant map file | No | No | Yes | No |
| Allele frequency data | No | No | No | Yes |
| Functional consequence information | No | No | No | Yes |
| Transposed plot | No | No | No | Yes |
| Additional SNP information (Odds Ratio, p-values) on the plot | No | No | No | Yes |
